# Trimethylamine *N*‐oxide is elevated in postmenopausal women relative to age‐matched men and premenopausal women among individuals with obesity

**DOI:** 10.1113/EP092550

**Published:** 2025-07-20

**Authors:** Daniel J. Battillo, Steven K. Malin

**Affiliations:** ^1^ Department of Kinesiology and Health Rutgers University New Brunswick New Jersey USA; ^2^ Division of Endocrinology, Metabolism and Nutrition Rutgers University New Brunswick New Jersey USA; ^3^ New Jersey Institute for Food, Nutrition and Health Rutgers University New Brunswick New Jersey USA; ^4^ Institute of Translational Medicine and Science Rutgers University New Brunswick New Jersey USA

**Keywords:** aortic waveforms, cardiovascular disease, central haemodynamics, glucose metabolism, menopause, sex differences, TMAO, trimethylamine *N*‐oxide

## Abstract

Trimethylamine *N*‐oxide (TMAO) is linked to arterial stiffness and atherosclerosis. Cardiovascular disease (CVD) risk increases following menopause in women. Whether menopause influences plasma TMAO metabolism to mediate CVD risk is unknown. Women with obesity were classified as premenopausal (*n* = 13; 40.3 ± 2.7 years; 39.4 ± 2.0 kg/m^2^) or postmenopausal (*n* = 22; 56.5 ± 1.1 years; 35.6 ± 0.9 kg/m^2^) via self‐reported presence/absence of menses (last 12 months). Men were age‐ and body mass index‐matched to postmenopausal women (*n* = 16; 55.9 ± 2.1 years; 34.3 ± 1.2 kg/m^2^) as controls to discern potential menopause‐driven TMAO differences. Carotid–femoral pulse wave velocity (cfPWV) and pulse wave analysis (applanation tonometry) were analysed to assess arterial stiffness, aortic waveforms and blood pressure. Fasting plasma TMAO and precursors (carnitine, choline, betaine and trimethylamine (TMA)) were assessed (mass spectroscopy). A 180 min 75 g oral glucose tolerance test was performed to approximate insulin sensitivity and quantify vascular cell (vascular cell adhesion molecule 1 (VCAM‐1)) and intercellular adhesion molecules (intercellular adhesion molecule 1 (ICAM‐1)). Body composition (DXA/BodPod) and fitness (${\dot{V}}_{O_{2}\mathrm{peak}}$) were measured. Premenopausal women were younger than men and postmenopausal women (*P *< 0.0001, η^2^ = 2.29). Men had lower body fat (*P *= 0.001, η^2^ = 0.80) and higher fat‐free mass (*P *= 0.004, η^2^ = 0.42) compared to both pre‐ and postmenopausal women. There were no differences among groups in fitness, insulin sensitivity, ICAM‐1 or blood pressure (*P *> 0.05), but men had higher cfPWV (*P *= 0.040, η^2^ = 0.27) and VCAM‐1 (*P *= 0.041, η^2^ = 0.32). Postmenopausal women had elevated TMAO (*P *= 0.040, η^2^ = 0.29), compared with men and premenopausal women, yet men had elevated TMA (*P *= 0.041, η^2^ = 0.17), carnitine (*P *= 0.003, η^2^ = 0.27), choline (*P *= 0.022, η^2^ = 0.35) and betaine (*P *< 0.0001, η^2^ = 0.59). Thus when taken together, menopause may raise TMAO in women, while older men appear to have unique TMAO precursor metabolism linked to CVD risk.

## Abstract (highlights)

- **What is the central question of this study?**
  Trimethylamine *N*‐oxide (TMAO) is related to arterial stiffness and atherosclerosis in ageing adults. But, does TMAO metabolism differ by menopause in women or by sex to promote cardiovascular disease (CVD) risk.
- **What is the main finding and its importance?**
  Postmenopausal women with obesity had elevated fasting plasma TMAO levels relative to age‐ and body mass index‐matched men and premenopausal women, independent of aerobic fitness, body fat and fat free mass. However, men had greater plasma TMAO precursors and elevated arterial stiffness. This suggests menopause may alter TMAO metabolism in women, despite sex‐specific effects between men and women relating to differences in CVD risk.

## 1 INTRODUCTION

Cardiovascular disease (CVD) is a leading cause of death in the United States (Ahmad & Anderson, 2021). Although women tend to have a lower risk for CVD in earlier life stages than men, the lifetime risk of CVD is similar (Woodward, 2019). This increased CVD risk among ageing women has been attributed to a loss of oestrogen at the onset of menopause (Boese et al., 2017; Iorga et al., 2017). In fact, oestrogen is thought to be so important that it contributes to cardioprotective effects in men as well (Cooke et al., 2017), and older men (e.g. 70 years old), who maintain their ambient oestrogen levels through ageing (Greenblatt et al., 1976), have lower CVD risk than younger, postmenopausal women (e.g. 50 years old) (Rodgers et al., 2019). Not surprisingly, cessation of ovarian oestrogen production in postmenopausal women has been linked to myriad CVD risk factors such as hyperglycaemia, dyslipidaemia, and hypertension (Prabakaran et al., 2021; Ryczkowska et al., 2022). Moreover, oestrogen promotes vascular health through the stimulation of endothelial derived nitric oxide which, in turn, favours vascular function and blood flow, reduces incidence of ischemia (Chambliss & Shaul, 2002; McNeill et al., 2002; Mendelsohn & Karas, 1999), and promotes the regulation of endothelial cell apoptosis and inflammation (Khalil, 2013; Meng et al., 2021). In addition to CVD risk among ageing women being attributed to the loss of oestrogen‐mediated benefits on the vasculature, alterations in gut microbiota following menopause have been implicated as a source of oestrogen, thereby showcasing a highly complex interaction among these factors between sexes (Novakovic et al., 2020).

Trimethylamine *N*‐oxide (TMAO) is a gut‐derived metabolite of particular interest in atherosclerosis development. TMAO is oxidized in the liver from trimethylamine (TMA), which is a metabolite produced in the gut from dietary nutrients like choline, carnitine and betaine that are found in meat, poultry, fish and eggs (Velasquez et al., 2016). Notably, TMAO has been implicated in vascular remodelling and arterial stiffness through increased foam cell production and inflammation (Wang et al., 2021; Zhu et al., 2020), and lifestyle modification can reduce TMAO in parallel with improved cardiovascular health (Erickson et al., 2019; Steele et al., 2021). Indeed, hepatic insulin resistance has been proposed as a linkage between obesity and elevated TMAO levels (DiNicolantonio et al., 2019). Therefore, lifestyle interventions that lower body fat and/or decrease insulin resistance may be successful in both reducing plasma TMAO concentrations and improving cardiovascular health. However, it is unknown if circulating TMAO concentrations differ by menopausal status in relation to CVD risk. Given that both ageing is linked to weight gain and TMAO has been reported to be elevated among individuals with obesity (Barrea et al., 2018; Schugar et al., 2017), we sought to test the hypothesis that TMAO and related precursors would differ among postmenopausal women in comparison to age‐matched men and premenopausal women with obesity. We secondarily looked to determine if TMAO or related precursors are related to arterial stiffness, aortic waveforms and/or haemodynamics as indices of CVD risk.

## 2 METHODS

### 2.1 Participants

Thirty‐five female participants and sixteen male participants with obesity were recruited for this cross‐sectional secondary analysis using newspaper flyers, social media advertisements and/or electronic medical records from the Charlottesville, VA and New Brunswick, NJ areas. Participants were classified as either premenopausal or postmenopausal based on the self‐reported presence versus natural absence of a regular menstrual cycle for at least 12 months. Those who reported as perimenopausal or having an irregular menstrual cycle were excluded as well as those reporting oral contraceptive or hormone replacement use. Older men were matched with postmenopausal women based on age and body mass index (BMI). Individuals were included in the present study if non‐smoking, physically inactive (<60 min/week), weight stable within 2 kg over the prior 6 months, not pregnant or nursing, and not on medications known to influence glucose metabolism (e.g. biguanides, insulin, SGLT‐2 inhibitors, etc.). All individuals had undergone fasted bloodwork (e.g. blood lipids, HbA1c, etc.) and physical examination along with resting/exercise EKG work to confirm the absence of chronic disease (e.g., hepatic, cardiovascular, renal, etc.) or cardiac abnormalities. Participants provided written and verbal informed consent prior to participation as part of a larger clinical trial (no. NCT03355469). Study protocols conformed to the *Declaration of Helsinki* and were approved by the Institutional Review Board (IRB nos 18316, 19364 and 2020002029). Subsequently, participants were pooled across different studies to test the current study purpose, resulting in differences in body composition as well as fitness assessments (see below).

### 2.2 Body composition

Total body weight was recorded to the nearest 0.1 kg on a digital scale (DS5100, Doran Scales, St Charles, IL, USA) with participants wearing minimal clothing and removing their shoes. Waist circumferences were measured in duplicate with a tape measure placed approximately 2 cm above the umbilicus and averaged. Height was measured to the nearest 0.1 cm using a stadiometer, and BMI was calculated. Total fat and fat free mass were measured via dual‐energy X‐ray absorptiometry (DXA) (premenopausal *n *= 4, postmenopausal *n *= 10, and men *n *= 16; Horizon DXA System; Hologic, Marlborough, MA, USA) or via air displacement plethysmography (premenopausal *n *= 9 and postmenopausal *n *= 12; BodPod, Concord, CA, USA), following a 4 h fast (food and beverage, except water) and minimal physical activity period before testing as previously indicated by our group (Gilbertson et al., 2019; Malin et al., 2022). These measurement modalities have been demonstrated to provide statistically similar measures of body composition among male and female adults with obesity (Nickerson et al., 2020).

### 2.3 Aerobic fitness testing

Participants completed an incline graded incremental treadmill (premenopausal *n *= 4, postmenopausal *n *= 10, and men *n *= 16) (Battillo et al., 2024) or cycle ergometer test (premenopausal *n *= 9 and postmenopausal *n *= 12) (Heiston et al., 2021) with indirect calorimetry to assess respiratory gases and peak oxygen consumption as previously completed by our group. Heart rate (HR) and rating of perceived exertion (RPE) were also recorded.

### 2.4 Oral glucose tolerance test

Participants arrived at the Clinical Research Center after an approximate 10 h overnight fast. Participants were also instructed to consume mixed‐meal diets (e.g. 55% carbohydrates, 30% fat and 15% protein), and food logs were collected to confirm intake. Further, habitual diet for 3 days was obtained to assess overall dietary caloric and macronutrient intake using The Food Nutrition Processor Analysis Software as previously done by our group (version 11.1; ESHA Research, Salem, OR, USA) (Gilbertson et al., 2019). Individuals were instructed to refrain from caffeine, alcohol, medication and strenuous exercise in the 24 h prior to each study visit. An intravenous catheter was placed in the right antecubital fossa for fasting blood draws as well as to determine glucose and insulin responses to a 75 g oral glucose load. Blood draws were thus collected at 0, 30, 60, 90, 120 and 180 min of the test to calculate glucose tolerance and whole‐body insulin sensitivity, which was estimated using the Matsuda Index (Matsuda & DeFronzo, 1999). Fasting insulin resistance was also estimated using the homeostatic model assessment of insulin resistance (HOMA‐IR). Fasting blood samples were collected to measure TMAO, TMA, carnitine, betaine and choline. Total area under the curve (tAUC) was calculated using the trapezoidal model for OGTT analytes. Metabolic syndrome ATP III risk factor scores were calculated to assess CVD risk. Aortic waveforms and arterial stiffness were measured at baseline as well.

### 2.5 Pulse waveform analysis

Fasting aortic waveforms and haemodynamics were characterized using applanation tonometry via the SphygmoCor XCEL system (AtCor Medical, Itasca, IL, USA). Briefly, measurements were collected with participants lying quietly in the supine position in a temperature‐controlled room after 10 min of rest. Pulse wave analysis outcomes included brachial systolic (bSBP), diastolic (bDBP) and pulse pressure (bPP), central systolic (cSBP), diastolic (cDBP) and pulse pressure (cPP), mean arterial pressure (MAP), heart rate (HR), augmentation pressure (AP) and index (AIx) as well as wave convolution aspects of forward pressure (Pf), backward pressure (Pb), and reflection magnitude (RM). Pulse pressure amplification (PPA) was calculated as the ratio of bPP to cPP. The augmentation index was corrected to a standard HR of 75 bpm (AIx75).

### 2.6 Pulse wave velocity

Fasting carotid–femoral pulse wave velocity (cfPWV) was assessed using applanation tonometry (AtCor Medical) to assess arterial stiffness. Participants remained in the supine position following pulse wave analysis. The carotid pulse was identified via palpation, and a tape measure was used to measure the length from the carotid pulse to a blood pressure cuff placed on the left upper thigh. The distance from the carotid pulse to the sternal notch was measured to account for parallel transmission in the aorta and carotid artery. Carotid pulse waves were assessed via the tonometer, and femoral pulse waves were assessed by the inflated blood pressure cuff on the thigh. cfPWV was calculated as the distanced travelled by the pulse wave divided by the pulse transit time. Analyses of cfPWV were covaried by mean arterial pressure as recommended by American Heart Association guidelines (Townsend et al., 2015).

### 2.7 Biochemical analysis

Plasma glucose was measured in duplicate immediately after collection via the glucose oxidase method (YSI Instruments 2300, Yellow Spring, OH, USA). Blood samples were collected in EDTA tubes containing aprotinin and centrifuged at 4°C for 10 min at 1500 *g*. Samples were frozen at −80°C until further analysis. Plasma samples of insulin (Millipore, Billerica, MA, USA) and vascular cell adhesion molecule 1 (VCAM‐1)/intercellular adhesion molecule 1 (ICAM‐1) (Bio‐Techne, Minneapolis, MN, USA) were analysed in duplicate using an ELISA. Plasma betaine, choline, carnitine, TMA and TMAO were analysed via liquid chromatography–tandem mass spectrometry as described previously by our group (Battillo & Malin, 2023).

### 2.8 Statistical analysis

Data were analysed using SPSS (V. 28.0, IBM Corp., Armonk, NY, USA) and GraphPad Prism Version 9 (GraphPad Software, Boston, MA, USA). Normality was assessed via the Shapiro–Wilk test, and non‐normally distributed data were log‐transformed for analysis. Differences among groups were analysed using a one‐way ANCOVA controlling for aerobic fitness relative to body weight, body fat and fat free mass. If there were group differences, a *post hoc* analysis was completed using Tukey's honestly significant difference (HSD) test. Partial eta squared was calculated to assess physiological relevance among group differences, which was interpreted as small η^2^ = 0.01, medium η^2^ = 0.06, and large η^2^ = 0.14. Pearson or Spearman's rank correlation was used to assess normally and non‐normally distributed outcomes, when appropriate. Statistical significance was accepted as *P* ≤ 0.05 and data are presented as means ± SEM.

## 3 RESULTS

### 3.1 Participant characteristics

There were no differences among groups in weight (*P *= 0.091, η^2^ = 0.10), yet men had a lower body fat percentage (*P *= 0.001, η^2^ = 0.80) and higher fat free mass (*P *= 0.004, η^2^ = 0.42; Table 1) than the pre‐ and postmenopausal groups. Although men had higher aerobic fitness relative to body weight (*P *= 0.003, η^2^ = 0.39), this difference was no longer significant when fitness was normalized to fat free mass (*P *= 0.081, η^2^ = 0.11; Table 1). Additionally, there were no differences among groups in ATP III score, fasting, 120 min, or tAUC glucose, insulin or HbA1c (*P *> 0.05; Table 1). Groups were similar as well regarding HOMA‐IR (*P *= 0.682, η^2^ = 0.03) and whole‐body insulin sensitivity (*P *= 0.701, η^2^ = 0.02; Table 1). There were also no differences in habitual dietary intakes among groups as indicated by similar total caloric (*P *= 0.144, η^2^ = 0.12), carbohydrate (*P *= 0.391, η^2^ = 0.05), fat (*P *= 0.192, η^2^ = 0.09) and protein (*P *= 0.091, η^2^ = 0.13; Table 1) intake.

**TABLE 1:** Participant characteristics.

	Premenopausal	Postmenopausal	Men	ANOVA	η^2^
Demographics					
*n*	13	22	16	—	—
Non‐Hispanic white	7	19	13	—	—
Non‐Hispanic black	4	1	1	—	—
Hispanic	—	2	1	—	—
Asian Pacific Islander	2	—	1	—	—
Age (years)	40.3 ± 2.7^*^	56.5 ± 1.1	55.9 ± 2.1	<0.0001	2.29
Anthropometrics					
Weight (kg)	112.1 ± 6.1	96.9 ± 3.2	106.1 ± 4.7	0.091	0.10
BMI (kg/m^2^)	39.4 ± 0.9	35.6 ± 2.0	34.3 ± 1.2	0.132	0.12
Body fat (%)	47.8 ± 2.7	48.6 ± 1.1	35.9 ± 1.2^*^	0.001	0.80
FFM (kg)	58.8 ± 2.4	51.4 ± 1.4	68.0 ± 2.8^*^	0.004	0.42
Fitness					
${\dot{V}}_{O_{2}\mathrm{peak}}$ (mL/kg/min)	20.4 ± 1.5	19.3 ± 0.8	26.8 ± 1.2^*^	0.003	0.39
${\dot{V}}_{O_{2}\mathrm{peak}}$ (mL/kg‐FFM/min)	37.5 ± 1.9	36.6 ± 1.4	41.2 ± 1.9	0.081	0.11
Cardiometabolic disease risk					
ATP III score	2.9 ± 0.2	3.1 ± 0.2	3.3 ± 0.2	0.423	0.04
WC (cm)	116.1 ± 5.2	110.1 ± 1.9	115.8 ± 3.4	0.321	0.05
TG (mmol/L)	118.3 ± 18.3	141.6 ± 16.8	146.6 ± 23.9	0.634	0.02
HDL‐c (mmol/L)	42.8 ± 1.8	48.9 ± 1.9	43.8 ± 2.8	0.321	0.05
Glycaemia					
Fasting (mg/dL)	101.0 ± 4.8	101.2 ± 1.6	98.6 ± 2.5	0.771	0.01
120 min (mg/dL)	131.1 ± 10.3	123.0 ± 5.4	134.1 ± 6.9	0.482	0.03
tAUC_180min_ (mg/dL)	23662 ± 1971	23101 ± 647	24928 ± 1105	0.521	0.03
HbA1c (%)	5.5 ± 0.1	5.5 ± 0.1	5.7 ± 0.0	0.824	0.09
Insulin metabolism					
Fasting (µU/mL)	17.8 ± 2.5	16.9 ± 2.8	14.1 ± 2.1	0.653	0.02
120 min (µU/mL)	93.4 ± 18.9	101.4 ± 14.3	85.0 ± 9.7	0.691	0.02
tAUC_180min_ (µU/mL)	14926 ± 2272	16302 ± 1948	14742 ± 2181	0.841	0.00
HOMA‐IR	4.4 ± 0.4	4.0 ± 0.7	3.5 ± 0.6	0.682	0.03
Matsuda index	3.5 ± 1.2	3.0 ± 0.3	3.6 ± 0.7	0.701	0.02
Liver and kidney function					
ALP (IU/L)	58.9 ± 4.4	65.9 ± 5.3	64.1 ± 4.0	0.382	0.04
AST (IU/L)	25.7 ± 2.3	29.8 ± 3.3	25.5 ± 1.8	0.282	0.05
ALT (IU/L)	29.6 ± 4.4	33.5 ± 5.2	34.1 ± 3.3	0.501	0.03
eGFR (mL/min/1.73)	90.1 ± 6.0	84.0 ± 3.5	88.4 ± 4.0	0.223	0.04
Dietary intake					
Total calories (kcal)	2222.8 ± 211.8	1903.4 ± 115.5	2181.8 ± 227.1	0.144	0.02
Carbohydrates (g)	242.3 ± 34.7	214.0 ± 15.1	244.1 ± 29.0	0.391	0.05
Fat (g)	92.6 ± 9.9	80.5 ± 6.9	94.6 ± 11.5	0.192	0.09
Protein (g)	106.3 ± 16.9	79.1 ± 7.0	91.6 ± 10.2	0.091	0.13

*Note*: Data are means ± SEM. Differences among groups were assessed via one‐way ANOVA and *post hoc* Tukey HSD test. ^*^ *P* ≤ 0.05, compared with other groups. Partial η^2^ was calculated.

Abbreviations: ALP, alkaline phosphatase; ALT, alanine transaminase; AST, aspartate transaminase; BMI, body mass index; eGFR, estimate glomerular filtration rate; FFM, fat free mass; HOMA‐IR, homeostatic model assessment of insulin resistance; ${\dot{V}}_{O_{2}\mathrm{peak}}$, peak aerobic capacity.

### 3.2 TMAO and precursors

**FIGURE 1:**
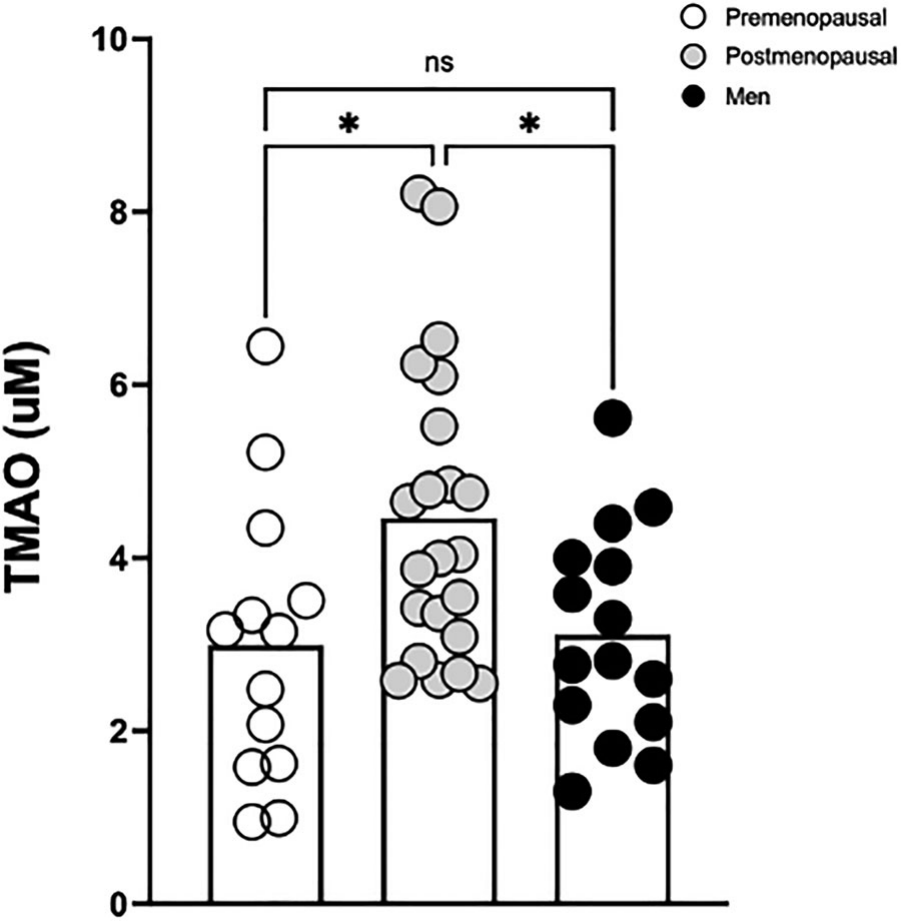
Fasting plasma TMAO levels among premenopausal women, postmenopausal women and men. Differences among groups were assessed via one‐way ANOVA and *post hoc* Tukey HSD test. *P ≤ 0.05, significant group difference.

**FIGURE 2:**
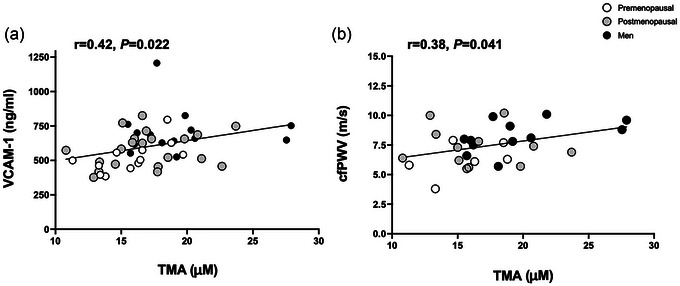
Correlation between fasting TMA and VCAM‐1 (a) and fasting TMA and cfPWV (b). White circles, premenopausal; grey circles postmenopausal; black circles, men.

Postmenopausal women had higher fasting TMAO than both premenopausal women and men (*P *= 0.040, η^2^ = 0.29; Figure 1), independent of group differences in aerobic fitness, body fat and fat free mass. Further, neither fitness relative to body weight (*r* = −0.20, *P *= 0.173), nor body fat (*r* = −0.28, *P *= 0.080) nor fat free mass (*r* = 0.22, *P *= 0.151) related to TMAO in the present study. Moreover, TMAO precursors TMA (*P *= 0.041, η^2^ = 0.17), carnitine (*P *= 0.003, η^2^ = 0.27), choline (*P *= 0.022, η^2^ = 0.35) and betaine (*P *< 0.0001, η^2^ = 0.59; Table 2) were all elevated among the men compared with both female groups, independent of aerobic fitness and body composition. Interestingly, higher fasting TMA related to both elevated VCAM‐1 (*r* = 0.42, *P *= 0.022; Figure 2a) and elevated cfPWV (*r* = 0.38, *P *= 0.041; Figure 2b), yet there were no significant relationships between TMA and insulin sensitivity or other vascular measures (data not shown). There was also no relationship between TMA and fitness (*r* = 0.21, *P *= 0.148), body fat (*r* = 0.01, *P *= 0.932) or fat free mass (*r* = 0.05, *P *= 0.720).

**TABLE 2:** Fasting plasma trimethylamine *N*‐oxide precursors.

	Premenopausal	Postmenopausal	Men	ANOVA	η^2^
TMA (µM)	15.5 ± 0.7	17.2 ± 0.7	19.3 ± 1.0^*^	0.041	0.17
Carnitine (µM)	34.2 ± 1.8	38.6 ± 1.6	44.6 ± 2.3^*^	0.003	0.27
Choline (µM)	11.1 ± 1.7	14.7 ± 1.5	17.9 ± 2.0^*^	0.022	0.35
Betaine (µM)	26.0 ± 2.6	31.6 ± 1.9	46.0 ± 2.4^*^	<0.0001	0.59

*Note*: Data are means ± SEM. Differences among groups were assessed via one‐way ANOVA and *post hoc* Tukey HSD test. ^*^ *P* ≤ 0.05, compared with other groups. Partial η^2^ was calculated. Abbreviation: TMA, trimethylamine.

### 3.3 Haemodynamics and arterial stiffness

There were no differences among groups in measures of central or peripheral blood pressure as well as AIx75 (*P *= 0.729, η^2^ = 0.03), AP (*P *= 0.342, η^2^ = 0.05) and other aortic waveform outcomes (*P *> 0.05; Table 3). However, men had elevated cfPWV, compared to the pre‐ and postmenopausal women (*P *= 0.040, η^2^ = 0.27; Table 3), independent of MAP. Additionally, while there were no differences among the groups in ICAM‐1 (*P *= 0.104, η^2^ = 0.13), men displayed elevated VCAM‐1 (*P *= 0.041, η^2^ = 0.32; Table 3).

**TABLE 3:** Fasting aortic waveforms, arterial stiffness and inflammatory markers.

	Premenopausal	Postmenopausal	Men	ANOVA	η^2^
Blood pressure					
bSBP (mmHg)	129.2 ± 3.7	139.6 ± 4.5	140.5 ± 4.6	0.668	0.10
bDBP (mmHg)	78.4 ± 2.2	83.5 ± 2.5	85.9 ± 2.5	0.562	0.12
cSBP (mmHg)	118.8 ± 2.9	129.4 ± 4.2	130.3 ± 4.1	0.551	0.09
cDBP (mmHg)	76.2 ± 4.4	84.8 ± 2.6	86.9 ± 2.5	0.694	0.07
MAP (mmHg)	95.3 ± 2.5	102.2 ± 3.1	104.1 ± 3.1	0.138	0.08
bPP (mmHg)	50.8 ± 2.6	56.2 ± 2.6	54.5 ± 3.0	0.541	0.04
cPP (mmHg)	38.8 ± 1.4	44.3 ± 2.2	43.4 ± 2.4	0.251	0.08
PPA (a.u.)	1.3 ± 0.0	1.3 ± 0.0	1.3 ± 0.0	0.568	0.03
HR (bpm)	64.8 ± 2.4	64.4 ± 2.0	62.6 ± 2.5	0.583	0.01
Arterial stiffness					
cfPWV (m/s)	6.2 ± 0.5	7.4 ± 0.5	8.3 ± 0.4^*^	0.040	0.27
Aortic waveforms					
AIx75 (%)	29.4 ± 2.8	29.1 ± 3.1	28.5 ± 3.3	0.729	0.03
AP (mmHg)	12.2 ± 1.6	15.8 ± 1.9	15.3 ± 1.8	0.342	0.05
Pf (mmHg)	27.6 ± 1.2	28.9 ± 1.7	26.8 ± 1.5	0.141	0.12
Pb (mmHg)	16.7 ± 0.8	20.1 ± 1.1	20.4 ± 1.5	0.338	0.08
RM (%)	61.5 ± 3.7	70.7 ± 3.2	68.3 ± 2.3	0.772	0.04
Inflammation					
VCAM‐1 (ng/mL)	522.1 ± 32.3	583.4 ± 28.0	798.7 ± 98.6^*^	0.041	0.32
ICAM‐1 (ng/mL)	223.7 ± 17.8	257.0 ± 16.7	233.3 ± 16.9	0.104	0.13

*Note*: Data are mean ± SEM. Differences among groups were assessed via one‐way ANOVA and *post hoc* Tukey HSD test. ^*^ *P* ≤ 0.05, compared with other groups. Partial η^2^ was calculated. Abbreviations: bSBP, brachial systolic blood pressure; bDBP, brachial diastolic blood pressure; cSBP, central systolic blood pressure; cDBP, central diastolic blood pressure; MAP, mean arterial pressure; bPP,  brachial pulse pressure; cPP, central pulse pressure; PPA, pulse pressure amplification; HR, heart rate; cfPWV, carotid–femoral pulse wave velocity; AIx75, augmentation index corrected to 75 bpm (heart rate); AP, augmentation pressure; Pf, forward pressure; Pb, backward pressure; RM, reflection magnitude; VCAM‐1, vascular cell adhesion molecule 1; ICAM‐1, intracellular adhesion molecule 1.

## 4 DISCUSSION

Prior work highlights that older adults with overweight have elevated TMAO levels in line with heightened CVD risk (Fretts et al., 2022; Lee et al., 2021). However, these prior findings combined men and women, thereby confounding the ability to discern ageing and sex‐based influences on TMAO metabolism. The primary finding from this study is that postmenopausal women with obesity have higher fasting plasma TMAO than both age‐matched men and premenopausal women with obesity. However, men had elevated TMAO precursors TMA, choline, carnitine and betaine. These differences remained after controlling for groups differences in aerobic fitness, body fat and fat free mass. Further, men displayed greater cfPWV than both premenopausal and postmenopausal women. This highlights that menopause may alter TMAO metabolism in women with obesity, prior to the presentation of arterial stiffness, in comparison to age‐ and BMI‐matched men. In contrast, sex‐specific effects between men and women may promote differences in TMAO precursor metabolism that associate with CVD risk. For instance, men in the present study generally had a lower body fat percentage and higher aerobic fitness than pre‐ and post‐menopausal women. This is potentially important, given lower body fat and higher aerobic fitness are each associated with lower plasma TMAO levels (Argyridou et al., 2020; Mihuta et al., 2023). Yet, men had lower TMAO levels than postmenopausal women. This indicates fat mass and fitness are not primary drivers of TMAO precursor conversion to TMAO, which is supported by our results showing that neither fitness nor body fat correlated with circulating TMAO in this study. Further, these findings remained even after covarying for these differences in fitness and body fat. Therefore, ageing‐specific differences between men and women are likely to be at play to explain our TMAO metabolism findings. Of note, previous work among individuals with normal weight, excess weight and obesity has suggested that plasma TMAO levels of 5–10 µM raise CVD risk (Dannenberg et al., 2020; Lee et al., 2021; Papandreou et al., 2020). Herein, the average TMAO level among our postmenopausal women was approximately 4.5 µM, whereas premenopausal women and men averaged about 3 µM. Given that clinical markers of metabolic disease and CVD risk such as the ATP III criteria, glucose tolerance and insulin sensitivity were not significantly different among groups in this study, these data highlight that menopause, independent of ageing, may drive differences in circulating TMAO, compared with TMAO precursors, which are different from older men.

Elevated TMAO among postmenopausal women with obesity is likely mediated by multiple factors. The gut microbiome has become a central focus in TMAO metabolism, given its direct influence over conversion of dietary precursors to TMA that precede TMAO oxidation in the liver (Yang et al., 2019). Prior work in healthy, middle‐aged adults demonstrated that reductions in red meat, dairy, fish and eggs are effective in lowering plasma TMAO levels through reduced dietary consumption of TMAO precursors like choline, betaine and carnitine (Krüger et al., 2017; Wang, Luo et al., 2022). Interestingly, the gut microbiome undergoes changes during the transition through menopause (Park et al., 2023; Peters et al., 2022). In fact, while premenopausal women display greater gut microbiome diversity than age‐matched men, postmenopausal women have no differences compared with their male counterparts (Peters et al., 2022). Subsequently, it is important to consider whether the loss of oestrogen during menopause acts as a mediator of TMAO metabolism. We did not measure oestrogen in the current study, but FMO3 gene expression in the liver is elevated in young female versus male mice, and female mice demonstrate a positive correlation between oestrogen and FMO3 activity (Schugar & Brown, 2015). This is relevant, provided FMO3 is the enzyme responsible for TMA oxidation to TMAO in the liver (Chen et al., 2019; Shih et al., 2015) and consistent with other rodent studies reporting lower TMAO in males compared to females in conjunction with reduced hepatic FMO3 expression (Bennett et al., 2013; Miao et al., 2015). Interestingly, however, our work would appear not to support the idea that oestrogen increases FMO3 activity and TMAO oxidation, given postmenopausal women have elevated TMAO compared with their premenopausal counterparts, and we did not see differences between premenopausal women and older men. Nonetheless, while additional work on the role of oestrogen in liver metabolism of TMAO is required, it is noteworthy that higher oestrogen levels are associated with favourable gut microbiome diversity (Baker et al., 2017; Siddiqui et al., 2022). This gut diversity among premenopausal women may reduce circulating TMAO through reduced conversion of carnitine, choline, and/or betaine to TMA. This is relevant, given chronic consumption of dietary TMAO precursors can lead to increased levels of TMA and TMAO (Pierce et al., 2021; Velasquez et al., 2016). In our study, despite similar caloric and macronutrient intake, men had significantly elevated TMAO precursors compared with postmenopausal women. Thus, diet per se is unlikely to explain the differential TMAO levels across our study cohorts, and it would seem factors related to menopause and sex impact TMAO metabolism. More work elucidating TMAO metabolism differences among men and women is merited, particularly as it relates to gut microbiota.

Hepatic insulin action regulates TMAO oxidation in the liver (DiNicolantonio et al., 2019; Miao et al., 2015). Specifically, FMO3 is suppressed by insulin action in the liver (Chen et al., 2019). Fasting glucose and insulin often reflect hepatic glucose regulation. If menopausal women had higher HOMA‐IR scores, it could be inferred that differences in hepatic insulin action influence the conversion from TMA to TMAO. Yet, we observed no differences in fasting glucose, HOMA‐IR, or whole body insulin sensitivity in the present study. Although we did not use the euglycaemic hyperinsulinaemic clamp with stable isotopes to elucidate the role of hepatic glucose metabolism, and our estimate of hepatic insulin resistance via HOMA‐IR may over‐ or underestimate differences in insulin action, we also did not detect differences in the liver enzymes alkaline phosphatase, aspartate transaminase and alanine transaminase. Taken together, we interpret these data to suggest that hepatic insulin resistance was likely not a major factor explaining TMAO differences.

TMAO has been suggested to raise CVD risk through, in part, promotion of arterial stiffness and subsequent atherosclerosis (Chen et al., 2019). Elevated TMAO in the circulation is linked to endothelial dysfunction through increased foam cell production, inflammation and advanced glycation end‐products (AGEs) (Weber et al., 2022; Zhu et al., 2020). Previous literature has also reported associations between fasting TMAO levels and brachial/systemic blood pressure, where each 5 µM increase in TMAO is related to a 9% increase in hypertension risk (Wang, Tang et al., 2022). While blood pressure was similar across study cohorts in the present study, it is important to note that men had higher arterial stiffness despite having lower plasma TMAO levels. At first this may seem paradoxical since women often experience increased arterial stiffness after menopause (Chambliss & Shaul, 2002; McNeill et al., 2002; Mendelsohn & Karas, 1999). However, the time course for developing arterial stiffness is unclear. In fact, it has been suggested that ageing women may not see equivalent, or greater, arterial stiffness than men until much later in life (e.g. 70 years of age) (Ogola et al., 2018). Nonetheless, we were somewhat surprised by this observation, given TMAO has been linked to vascular inflammation as a driver of arterial stiffness among healthy middle‐aged to older adults (Brunt et al., 2021). However, these individuals had higher plasma TMAO levels than individuals in the present study and were older in age. Taken together, this highlights the differential development of arterial stiffness across the lifetime between sexes, prior to older age, may mitigate the relationship between arterial stiffness and TMAO in the present study. In line with this, men herein had elevated TMAO precursors TMA, choline, carnitine and betaine in parallel with elevated VCAM‐1. The adhesion molecules VCAM‐1 and ICAM‐1 are physiologically relevant as they initiate endothelial inflammatory processes (Querio et al., 2023), and TMAO was reported to increase VCAM‐1 expression and upregulate monocyte adherence in human umbilical vein endothelial cells (Ma et al., 2017). Although we did not detect significant associations between TMAO and VCAM‐1, TMA correlated with both higher VCAM‐1 and higher cfPWV. Interestingly, TMA, but not TMAO, was demonstrated to promote vasoconstriction and disrupt kidney function among individuals with cardiovascular disease, demonstrating the vascular effects of TMA and TMAO may also occur independently of one another (Jaworska et al., 2019). Thus, among individuals with obesity, elevated TMA in older men, but not postmenopausal women, may play a role in arterial‐mediated CVD risk. This also aligns with prior work from our group demonstrating that reductions in TMA and carnitine were related to reduced post‐prandial aortic waveforms following 2 weeks of low calorie diets, with and without aerobic exercise (Battillo & Malin, 2023). Further work examining TMAO precursor‐mediated sex differences in CVD risk is needed.

This study has limitations that warrant consideration. The present investigation was among a modest sample size of premenopausal women, postmenopausal women and men with obesity. Thus, it is not possible to generalize findings to cohorts of lean individuals. Further, body composition was assessed using either the BodPod or DXA scan across groups, which may introduce some measurement variability. Nonetheless, recent work among individuals with obesity did not find significant differences in fat‐free mass and body fat measured by the BodPod and DXA (Nickerson et al., 2020). Aerobic fitness was also measured using both a treadmill walking assessment and a cycle ergometer. While these assessment modalities can lead to variability in maximal aerobic fitness assessments, we did not see differences in aerobic fitness among those tested by treadmill or cycle ergometer in the present study, nor did covarying for aerobic fitness measured using each test individually change any statistical outcomes. We also did not measure levels of oestrogen or other hormones (e.g. follicle‐stimulating hormone) between groups but instead used self‐reporting to identify menopause status. However, we excluded perimenopausal women as well as those with irregular menses to more confidently examine how the presence versus absence of oestrogen might impact TMAO levels. Further work should consider examining TMAO across pre‐, peri‐, and post‐menopause. Additionally, we did not control for menstrual cycle phase in premenopausal women when completing clinical testing, given previous work examining blood pressure, arterial stiffness, and aortic waveforms changes across the menstrual cycle did not note differences in these outcomes across the menstrual phases of premenopausal women (Eagan et al., 2022). Moreover, we did not delineate the duration of menopausal status using the Stages of Reproductive Aging Workshop approach, and it is possible that early (e.g. 0–5 years) versus later (>5 years) menopause could yield unique insight towards TMAO metabolism, given the understanding that endothelial function declines with menopause duration (Moreau et al., 2012). Lastly, elevated TMAO levels in postmenopausal women have been related to TMAO clearance in the kidney (Krueger et al., 2021; Randrianarisoa et al., 2016). Thus, differences in kidney function across groups may have impacted our data. However, there were no differences in eGFR as a clinical indicator of kidney function among groups. Future work, nonetheless, might consider how chronic kidney disease relates to TMAO metabolism and vascular dysfunction in postmenopausal women.

In conclusion, postmenopausal women with obesity had elevated TMAO levels compared to both age‐matched men and premenopausal women with obesity, independent of aerobic fitness and body composition. In contrast, older men had elevated levels of TMAO precursors TMA, carnitine, choline and betaine, when compared with pre‐ and post‐menopausal women. Further, higher TMA related to elevated VCAM‐1 and cfPWV. These findings suggest menopause influences TMAO in women with obesity, while ageing may promote sex specific differences in the conversion of TMAO precursors to TMAO in age‐ and BMI‐matched adults. Additional insight into TMAO‐related mechanisms is warranted to improve precision medicine approaches that prevent, treat and/or delay the onset of CVD among ageing women and men.

## AUTHOR CONTRIBUTIONS

Steven K. Malin conceptualized the work and design. Steven K. Malin and Daniel J. Battillo contributed to data collection and/or analysis. Daniel J. Battillo was mainly responsible for statistical analysis with support from Steven K. Malin All authors have read and approved the final version of this manuscript and agree to be accountable for all aspects of the work in ensuring that questions related to the accuracy or integrity of any part of the work are appropriately investigated and resolved. All persons designated as authors qualify for authorship, and all those who qualify for authorship are listed.

## CONFLICT OF INTEREST

None declared.

## Data Availability

Data are available upon reasonable request from the corresponding author.
